# Enhancing Hardness and Wear Performance of Laser Additive Manufactured Ti6Al4V Alloy Through Achieving Ultrafine Microstructure

**DOI:** 10.3390/ma13051210

**Published:** 2020-03-08

**Authors:** Yanqin Li, Lijun Song, Pan Xie, Manping Cheng, Hui Xiao

**Affiliations:** 1 State Key Laboratory of Advanced Design and Manufacturing for Vehicle Bodies, Hunan University, Changsha 410082, China; yanqinlihnu@126.com (Y.L.); ljsonghnu@126.com (L.S.); manpingcheng@126.com (M.C.); 2 Hunan Provincial Key Laboratory of Intelligent Laser Manufacturing, Hunan University, Changsha 410082, China; 3 Center of High Resolution Electron Microscopy, College of Materials Science and Engineering, Hunan University, Changsha 410082, China

**Keywords:** laser additive manufacturing, titanium alloy, microstructure, hardness, wear properties

## Abstract

Refining microstructure is an important issue for laser additive manufacturing (LAM) of titanium alloy. In the present work, the microstructures of LAM-fabricated Ti6Al4V alloy were refined using a low energy density with the combination of a small spot diameter, a low laser power, and a high scanning speed. The microstructure, hardness, wear performance, and molten pool thermal behavior of LAM-fabricated Ti6Al4V coatings were studied. The results show that the grain sizes of both prior β and α phases are strongly dependent on the cooling rate of the molten pool. The fine prior β grains and submicron-scale acicular α phases were obtained under a low energy density of 75 J mm^−2^ due to the high cooling rate of the molten pool. In addition, the as-fabricated Ti6Al4V sample with submicron-scale acicular α phase showed a very high hardness of 7.43 GPa, a high elastic modulus of 133.6 GPa, and a low coefficient of friction of 0.48. This work provides a good method for improving the microstructure and mechanical performance of LAM-fabricated Ti6Al4V alloy.

## 1. Introduction

Titanium and titanium alloys have been widely used in many industries, especially aerospace, energy, and biomedical industries, due to their high specific strength, excellent corrosion resistance, and good biocompatibility [1,2,3]. Laser additive manufacturing (LAM) is a transformative technology for the fabrication of near-net shape components [4,5,6], and it is becoming more and more popular for the fabrication or repair of titanium alloy components [7,8,9,10]. 

LAM with coaxial powder feeding generally uses a high-energy laser to simultaneously melt and deposit metal powders onto a substrate and then fabricates parts layer by layer. During the LAM process, the rapid cooling of the molten pool creates a high cooling rate, a steep temperature gradient, and a non-equilibrium solidification. These processing features generally lead to special microstructures and mechanical properties of LAM-fabricated parts, compared to conventional manufacturing techniques. LAM-fabricated titanium alloy is often distinguished by coarse, columnar prior β grains due to the high temperature gradients [11,12] and fine lamellar or lath-like microstructure within prior β grains as a result of the high cooling rate [13,14]. In addition, LAM-fabricated titanium alloys often exhibit high strength due to refined microstructure and low ductility due to the high level of residual stress [7,15,16,17,18]. 

Ti6Al4V is one of the most widely used titanium alloys. It has a typical duplex (α + β) structure. The mechanical properties, especially the hardness, are strongly dependent on the scale of the microstructure of LAM-fabricated Ti6Al4V [9,19]. A basket-weave substructure, with the width of α phase ranging from hundreds of nanometers to several microns, is commonly observed for LAM of Ti6Al4V. Wu et al. [20] reported that the high scanning speed is beneficial for decreasing the sizes of both α and β laths. Kelly et al. [21] showed that the average width of α lath of the LAM-fabricated coatings is about 1.12 µm, and the corresponding hardness is about 351 HV_0.3_. Similar work was also conducted by Lu et al. [22]; their work showed that the micron scale α phase (1.8–2.8 µm) corresponds to the low average hardness of 338 HV_0.5_. Brandl et al. [23] found that the hardness of 300–440 HV_0.1_ can be obtained by achieving the lamellar α phase with a width of 0.6–1.5 µm. However, there are few previous studies aimed at refining the microstructure to the submicron or even nanoscale to further improve mechanical properties. 

Ti6Al4V is a frequently used titanium alloy for biomedical implant applications due to its high specific strength, low density, and biocompatibility. However, the low hardness, high friction coefficient, and high wear rate restrict its applications under severe friction conditions, e.g., hip implants [24]. In addition, high hardness and good wear performance are also necessary for other industrial applications [25,26]. Many efforts have used LAM as a cladding technology to modify the surface properties of titanium alloys [27]. Ceramics (nitride, carbide, oxide) and cermet (Ti-TiC, Al-TiC) were cladded on titanium alloy substrate to improve hardness and wear performance [28]. Unfortunately, the surface ceramic layers tend to crack, due to the different physical and chemical properties between the coating materials and the substrate. Efforts were conducted to improve hardness and wear performance by refining the microstructure of titanium alloy. Chikarakara et al. [29] produced the acicular α structure for improving the surface microhardness of Ti6Al4V using laser surface modification. Gu et al. [30] enhanced the hardness and wear performance of LAM-fabricated commercially pure Ti by obtaining fine α phases. Bartolomeu et al. [31] obtained high hardness and wear resistance of Ti6Al4V using the selective laser melting process to achieve a refined microstructure. Nassar et al. [32] reduced the width of α lath and improved the hardness of LAM-fabricated Ti6Al4V, using a pulsing laser beam. Nevertheless, to the best of the authors’ knowledge, there are still no comprehensive studies focusing on the enhancement of hardness and wear performance through microstructural refinement of LAM-fabricated Ti6Al4V. Moreover, the relationships among processing parameters, thermal behavior, microstructure, and wear performance of LAM-fabricated Ti6Al4V need to be further investigated. 

In this work, submicron-scale acicular α/α′ phases in LAM-fabricated Ti6Al4V were obtained using the combination of a small spot diameter and a high scanning speed. As-fabricated Ti6Al4V samples showed an excellent combination of high hardness and good wear performance. Relationships among process, molten pool thermal behavior, microstructure, and mechanical properties of LAM-fabricated Ti6Al4V were investigated.

## 2. Materials and Methods 

**Figure 1:**
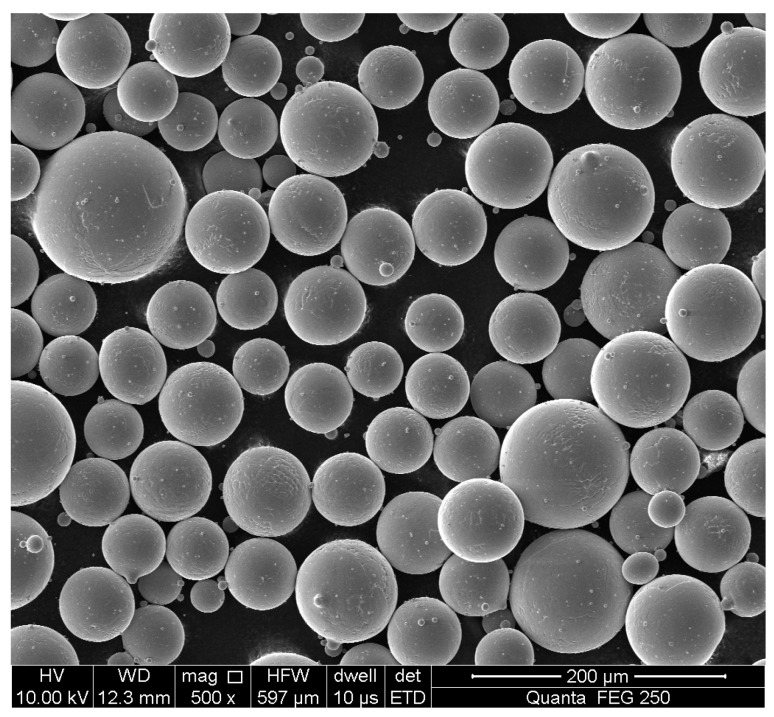
Morphology of Ti6Al4V powders.

**Figure 2:**
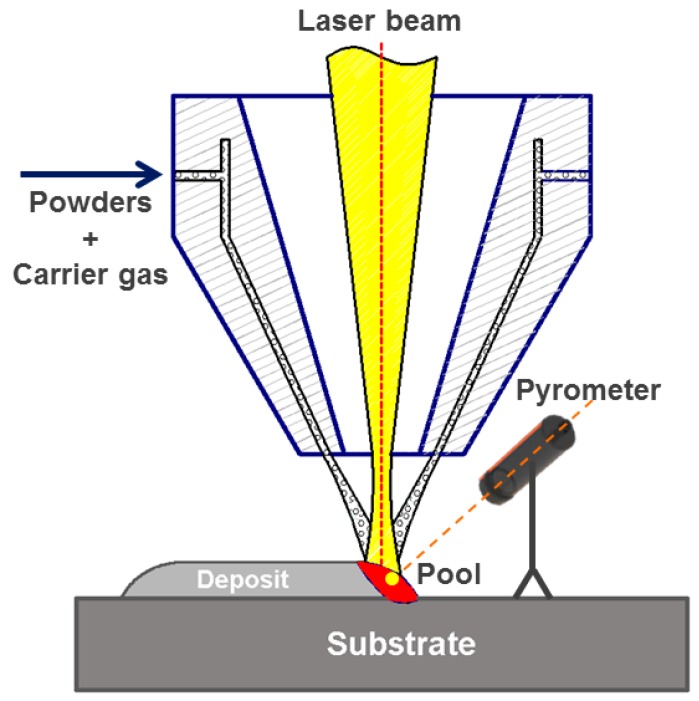
Schematic diagram showing the laser additive manufacturing (LAM) process.

LAM with coaxial powder feeding was used to fabricate Ti6Al4V samples on pure Ti plates. Gas-atomized Ti6Al4V powders were used. Figure 1 shows the typical morphologies of the Ti6Al4V powders. The average particle size is 50 µm, and 85% of the particles range from 40 to 90 µm. Two sets of samples were fabricated with different laser spot diameter *D*, laser power *P,* and scanning speed *V*. The sample fabricated at *D* = 3 mm, *P* = 1200 W, and *V* = 4 mm s^−1^ was defined as sample A, while the sample fabricated at *D* = 1 mm, *P* = 600 W, and *V* = 8 mm s^−1^ was defined as sample B. The other adopted processing parameters included powder feed rate of 12 g min^−1^ and shielding gas (argon) of 8 L min^−1^. A schematic diagram of the experimental setup is shown in Figure 2. 

The area energy density *E* (J mm^−2^) was used to evaluate the averaged energy input [33]:

(1) $$E=\frac{P}{V\times D}$$

where *P* is the laser power (in J s^−1^), *V* is the scanning speed (in mm s^−1^), and *D* is the laser spot diameters (in mm). Thus, two different “energy densities” (*E*) of 100 and 75 J mm^−2^ were used to evaluate the averaged laser energy input of sample A and sample B, respectively. 

During LAM, a two-color pyrometer was used to record the temperature of the molten pool in a noncontact way. The detailed setup parameters can be found in our previous works [4,34]. 

In this work, the single-track samples were used for microstructure and hardness characterization, and the multi-track specimens were used for wear tests. For multi-track samples, a 50% overlap ratio was adopted. Each multi-track sample has five-track depositions. The top section samples were prepared and etched by a Kroll’s etchant for 40 s. The microstructures of the fabricated samples were characterized using a Carl Zeiss Jena Axio Vert.A1 optical microscope (OM) and a FEI Quanta 250 FEG scanning electron microscope (SEM). The phase compositions of the samples were identified by a Rigaku-D/MAX-2550 micro-area X-ray diffractometer (XRD). The phase identification was performed using a JEM 2010 transmission electron microscope (JEM 2010). TEM specimens were prepared using a standard polishing procedure and a twin-jet electro-polishing technique using a solution of 21 vol% perchloric acid, 50 vol% methanol, and 29 vol% butyl alcohol.

Nano-hardness and elastic modulus of the as-fabricated samples were measured by a CSM UNHT nano-indentation tester with a Berkovich indenter. The applied test force was 300 mN, and hold time was 15 s. Three specimens in each condition were tested, and five nano-hardness measurements were carried out on each specimen. Linear reciprocating ball-on-block wear testing was performed using a UTM-3 tribometer. The ϕ3 mm hardened chrome steel ball (100Cr6, 58–63 HRC) was used as counterpart. All tests were performed with 5 N constant load, 10 mm linear oscillatory motion, 240 mm min^−1^ speed, and 40 min loading duration. Three specimens in each condition were tested to calculate the friction coefficient and the weight loss. All test results were calculated by averaging measurements. In addition, the values of the standard deviations were also given. A three-dimensional super-depth-of-field microscope was used to observe the worn surfaces of the samples under different processing parameters. 

## 3. Results

### 3.1. Phase Identification 

**Figure 3:**
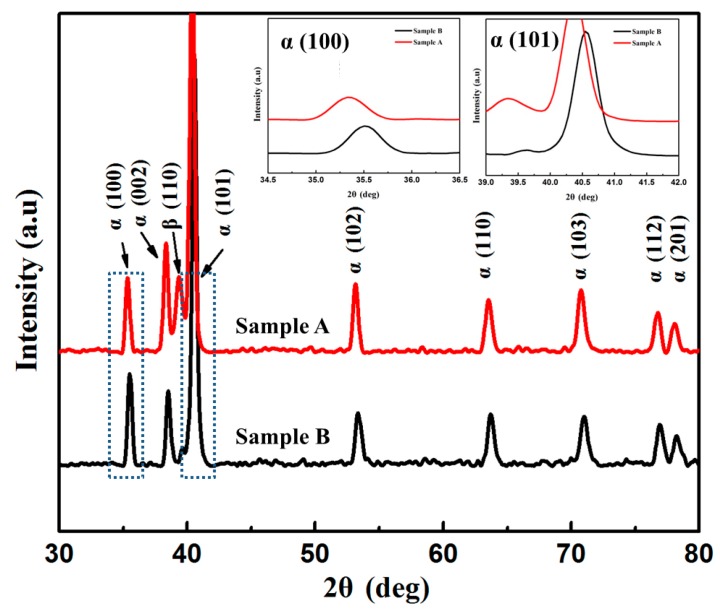
XRD patterns of the as-fabricated samples under different processing parameters.

Figure 3 shows the XRD patterns of the as-fabricated samples under different processing parameters. The diffraction peaks of the samples correspond to hcp-Ti (α phase) and bcc-Ti (β phase). The intensity of the β phase diffraction peak in sample A is higher than that of sample B, indicating higher contents of the β phase in the former. In addition, the diffraction peaks (100)α and (101)α shift toward higher angles in sample B (Figure 3). According to Bragg’s law, the observed increase of 2*θ* indicates a decrease in the lattice plane distance *d,* which is considered to be related to the prevailing martensitic transformation [30,35]. The occurrence of martensitic transformation is accompanied by microscopic volume expansion, which decreases the lattice parameters [36]. The high cooling rate during solidification of sample B is beneficial for promoting the martensitic transformation [37] and results in the shift of diffraction peaks (100)α and (101)α toward higher angles.

### 3.2. Microstructure of the as-Fabricated Samples

**Figure 4:**
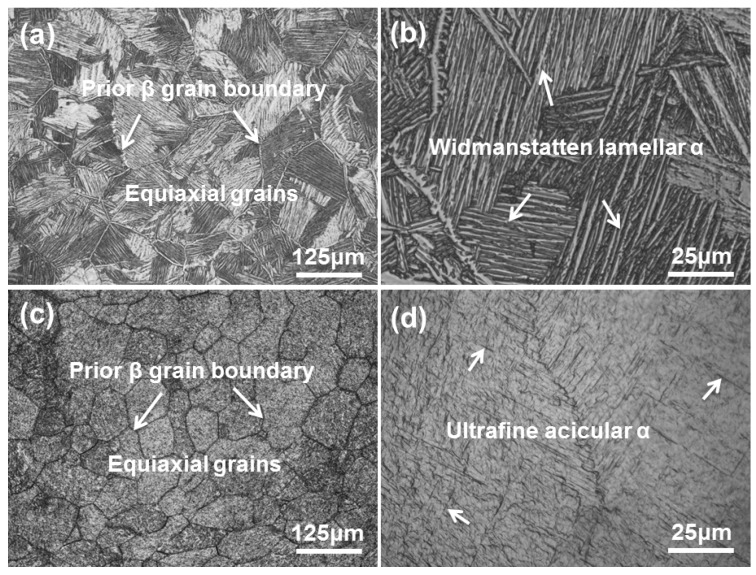
OM micrographs showing the top section microstructures of the fabricated samples: (**a**, **b**) sample A, and (**c**, **d**) sample B.

Figure 4 shows the OMs of as-fabricated Ti6Al4V samples under different processing parameters. The microstructure of sample A is dominated by equiaxed prior β grains (Figure 4a). The average grain diameter of the prior β grains is 135.5 µm. The substructure in prior β grains is composed of lath-like α phases, showing a typical Widmanstätten structure (Figure 4b). The average α lath width of sample A is 2.1 µm. Compared with sample A, sample B shows more refined prior β grains. The average diameter of prior β grains is 62.9 µm. Some lamellar α clusters can be observed at the prior β grain boundaries. More detailed features of the substructure in prior β grains should be further identified by SEM.

**Figure 5:**
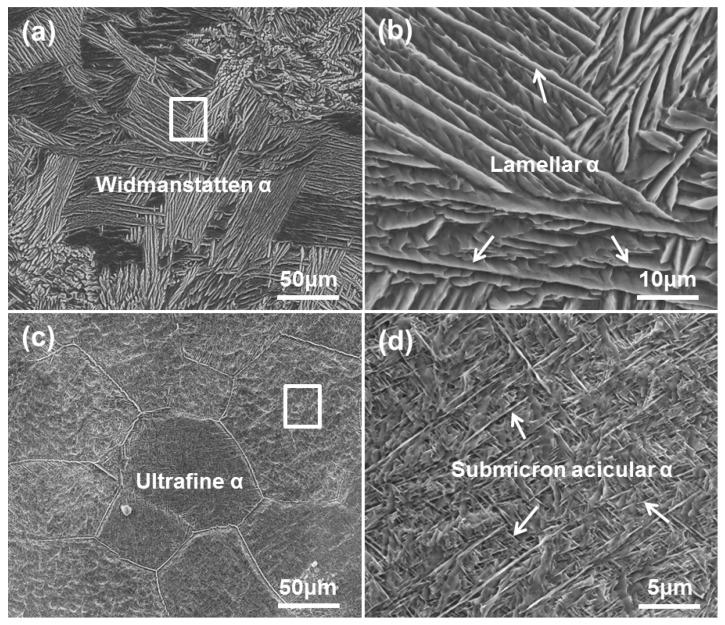
SEM micrographs showing the typical microstructures of fabricated Ti6Al4V samples: (**a**, **b**) sample A and (**c**, **d**) sample B.

Figure 5 shows the typical SEM morphologies of as-fabricated samples. The SEM micrographs are analogous to the OMs of as-fabricated samples in terms of prior β grain morphology (Figure 5a,c). Sample A is dominated by coarse lamellar α phases (Figure 5b). Compared with sample A, sample B shows finer equiaxed prior β grains in which submicron acicular α phases are embedded (Figure 5d). The thickness of the α phase is between 60 and 400 nm, which is smaller than that of 0.6–2.8 µm reported in the literature [20,22,23,38]. The ultrafine microstructure means that more excellent mechanical properties can be obtained.

**Figure 6:**
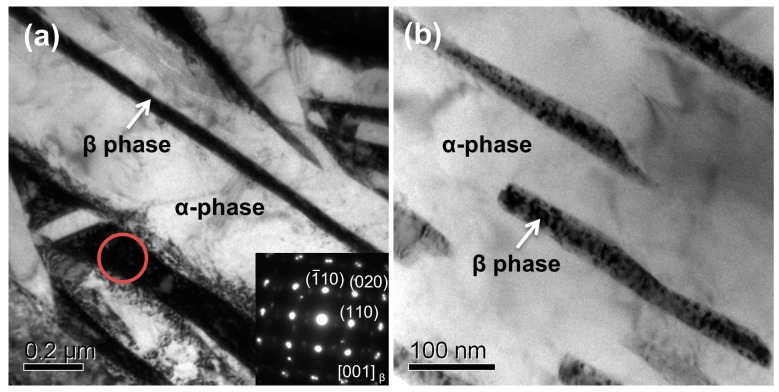
TEM analysis of the as-fabricated samples: (**a**) Bright-field (BF) TEM image showing the α phases and the retained β phases in sample A, and the insert showing the SAED pattern corresponding to β phase in the red circle; (**b**) BF TEM image showing the discontinuous β phases in sample B.

In order to further identify the structures of phases in the samples, TEM analysis was employed. Figure 6a,b indicates bright-field (BF) TEM micrographs of samples A and B, respectively. As shown in Figure 6a, a few of the lamellae in black contrast lay across fields of whole pictures. These lamellae are bcc-Ti (β phase) that is retained during the phase transformation of β→α. Inserted selected area electron diffraction (SAED) from the red circle in Figure 6a is indexed and indicates that the phases in black are identified as bcc-Ti (β phase). The average thicknesses of lamellae are estimated between 70 and 200 nm. However, both the amounts and sizes of lamellae in sample A are obviously larger than those of sample B (Figure 6b). The TEM observations are in accordance with the XRD results. 

### 3.3. Nano-hardness and Elastic Modulus

**Figure 7:**
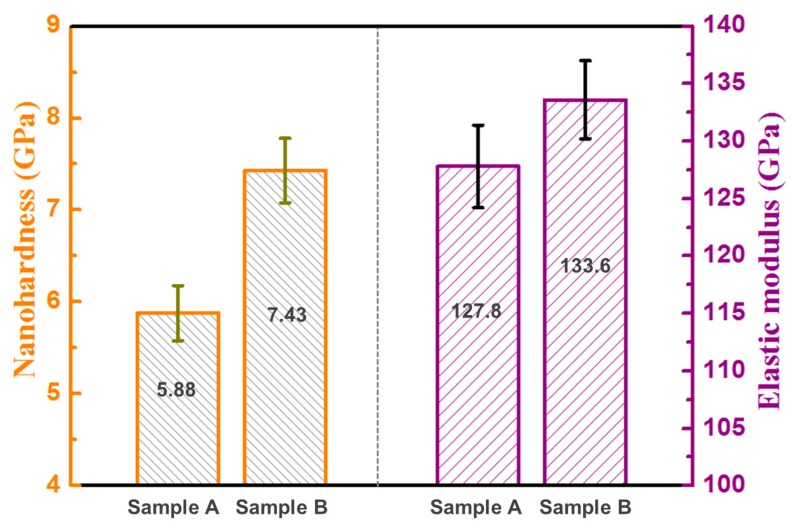
Nano-hardness (HIT) and elastic modulus (EIT) of the as-fabricated Ti6Al4V samples under different processing parameters.

Figure 7 shows the nano-hardness and the elastic modulus of the as-fabricated Ti6Al4V samples under different processing parameters. The average nano-hardness of sample B is 7.43 GPa, which is 26.3% higher than that of sample A (5.88 GPa). Compared to conventional powder-metallurgy-processed Ti6Al4, with a hardness of 3.77 GPa [39], the hardness of LAM-processed parts is much higher. The refined prior β grains and the obtained submicron acicular α phase contribute to the high nano-hardness of sample B. The average elastic modulus of sample B is 133.6 GPa, which is 4.5% higher than that of sample A (127.8 GPa). The elastic modulus of LAM-fabricated Ti6Al4V samples is much higher than that of a conventional powder-metallurgy-processed Ti6Al4V alloy at 110 GPa [40]. The high elastic modulus is supposed to be related to the high level of residual stress due to rapid solidification during the laser deposition process [41].

### 3.4. Wear Performance

**Figure 8:**
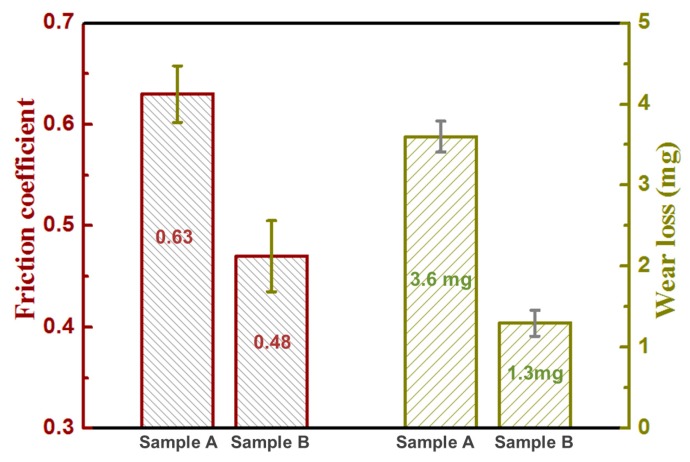
The friction coefficient and wear loss of the as-fabricated Ti6Al4V samples under different processing parameters.

Figure 8 shows the values of the friction coefficient and the wear loss of two samples. The friction coefficient of sample B is 0.48, which is lower than that of sample A (0.63). The wear loss of sample B is 1.3 mg, which is significantly less than that of sample A (3.6 mg). The reduction in friction coefficient and wear loss reveals the higher wear resistance of sample B. The improved wear resistance is associated with the ultrafine microstructure (formation of acicular α phase) and attended high hardness.

**Figure 9:**
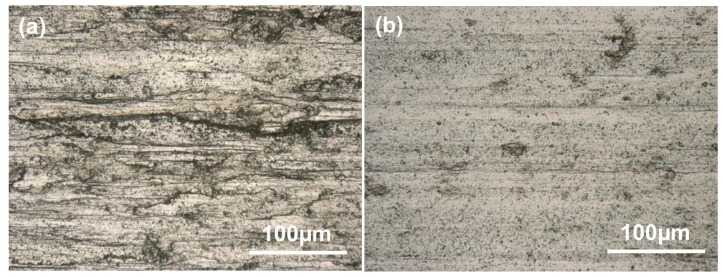
Optical micrographs indicating the morphologies of worn surfaces of the as-fabricated samples: (**a**) the sample A and (**b**) the sample B.

The worn surface of sample A is characterized by parallel and deep grooves, with some fragments on the groove edges, which indicate the occurrence of abrasion and fatigue wear (Figure 9a). During the back and forth wear tests, contact fatigue caused by alternating stress between the substrate and counterpart occurred, resulting in fragments that acted as abrasive particles. Compared to sample A, the worn surfaces of sample B were smoother. Shallower grooves and an almost total absence of any abrasive fragments were observed on the worn surfaces (Figure 9b), indicating that the main wear mechanism is slight fatigue and abrasive wear.

## 4. Discussion

### 4.1. Thermal Behavior of the Molten Pool

**Figure 10:**
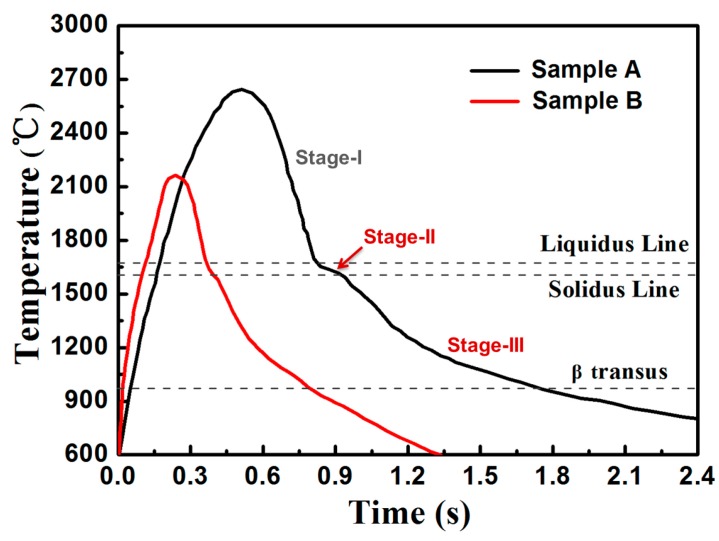
Thermal curves of the molten pool under two different processing conditions.

The solidification microstructure is related to the thermal behavior of the molten pool. Thus, the molten pool temperature was recorded and calculated to interpret relationships among the thermal behavior, processing parameters, and solidification microstructure. Figure 10 shows the thermal curves of the molten pool of two samples. The temperature of the measured point rapidly increased to the peak temperature with the approach of the laser beam and then rapidly cooled to the minimum temperature as the laser beam moved away. Compared to sample A, the temperature curve of sample B shows a lower peak temperature, a shorter thermal cycle, and a faster decrease in molten pool temperature. The cooling stage could be simply divided into three stages, i.e., stage I (cooling stage of liquid phase), stage II (solidification stage), and stage III (cooling stage of solid phase). Among these cooling stages, the solidification parameters of stage II determine the solidification process, while the thermal parameters of stage III influence the solid–state phase transition. 

For LAM of Ti6Al4V alloy, two kinds of phase transitions occur during the cooling stage of the molten pool: the transition of liquid phase to β phase during stage II and the transition of β phase to α phase during stage III. Thus, the average cooling rate and solidification time of molten pool during stage II were calculated to understand the formation of the solidified microstructure. The average cooling rate during stage III was estimated to comprehend the transition of β phase to α phase. The average cooling rate and the solidification time of the molten pool during stage II of sample A were 0.61 × 103 °C/s and 92 m/s, respectively, whereas a higher cooling rate of 2.53 × 103 °C/s, and a shorter solidification time of 22 m/s were obtained for sample B. In addition, the average cooling rate during stage III of sample B was 0.73 × 103 °C/s, which is higher than that of sample A (0.32 × 103 °C/s). The above results show that the low laser energy density enhances the cooling rate and decreases the solidification time of the molten pool.

### 4.2. Refinement of Microstructure

Compared with the large prior β grains and the coarse lath-like α phase in sample A, the more refined prior β grains and ultrafine acicular α phases were obtained in sample B. In addition, fewer retained β phases were observed in sample B. LAM-fabricated Ti6Al4V normally exhibits a martensitic microstructure and/or a Widmanstätten (or basket-weave) microstructure with the width of α phase ranging from 0.6 μm to several microns, such as the average width of α lath of 1.12 µm in [21], 1.8–2.8 µm in [22], and a lamellar α phase ranging from 0.6 to 1.5 µm in [23], whereas a more refined microstructure with submicron-scale acicular α phases was obtained in the present work. The observed microstructural difference was due to the different thermal behavior of the molten pool of the two samples. First, the low energy density with the combination of a small spot diameter, a low laser power, and a high scanning speed led to lower peak temperature, shorter thermal cycle, and less thermal accumulation of the molten pool. Thus, the quenching effect that was realized by thermal transmission through the substrate was strong. Second, the relatively low laser energy density and high scanning speed corresponded to the high cooling rate and high solidification rate of the molten pool, thereby increasing the undercooling of the solidification front. The significantly enhanced cooling rate and solidification rate refined microstructure and promoted phase transformation. Thus, the enhanced cooling rate and solidification rate during stage II (the transition of liquid phase to β phase) led to higher undercooling in the solidification front, which reduced the nucleation radius and increased the nucleation rate of β phase as well. Thus, the prior β grains were significantly refined for sample B. The formation of ultrafine acicular α phases depended on the cooling parameters of stage III (the transition of β phase to α phase). The high cooling rate during stage III promoted phase transition and the formation of ultrafine acicular α phases in sample B.

### 4.3. Enhancement of Hardness and Wear Performance

Sample B shows a high nano-hardness of 7.43 GPa, which is higher than that of sample A and conventional Ti6Al4V alloys. In addition, the hardness of sample B is also higher than that of results reported in the literature [21,22,23]. The high nano-hardness level is attributed to the refinement of β grains and the formation of the submicron acicular α phase. The increase of boundaries or interfaces enhanced the hindering effect on dislocation glide and therefore improved the hardness of sample B. In addition, the high level of residual stress in the as-fabricated samples also contributed to the high nano-hardness and elastic modulus.

The wear performance closely depends on the surface microstructure and mechanical properties of the coating. Compared to sample A, the prior β grains and α phase of sample B are finer, and the corresponding hardness is higher. Thus, the tribolayer of sample B can easily smear plastically on the surface being slid [42]. It is believed that the mode of material removal during sliding changes from abrasion to adhesion of the tribolayer with the decrease of laser energy input. Such a transition results in a reduction in wear loss during sliding. Therefore, it is reasonable to conclude that low energy density favors improvement in the wear performance of LAM-fabricated Ti6Al4V alloy. The energy density should be as low as possible, within reasonable range, to obtain an ultrafine microstructure and attendant high hardness, which is beneficial for enhanced wear performance of the LAM-fabricated Ti6Al4V alloy.

## 5. Conclusions

Ti6Al4V coatings were fabricated by LAM with different processing parameters. The microstructure, hardness, wear performance, and thermal behavior of the molten pool of two typical as-fabricated samples were investigated comparatively. The following conclusions can be drawn:

1. Compared to the high peak temperature, long thermal cycle, and low cooling rate of the molten pool obtained at high energy density with a low scanning speed, low energy density with a high scanning speed produced the shorter thermal cycle, higher cooling rate, and shorter solidification time of the molten pool.
2. An ultrafine microstructure with submicron-scale acicular α phase was achieved for sample B due to the high cooling rate and the short solidification time of the molten pool.
3. Compared to sample A, sample B showed a very high hardness of 7.43 GPa, a high elastic modulus of 133.6 GPa, a low coefficient of friction of 0.48, and a low wear loss of 1.3 mg.
4. The worn mechanism of sample A is abrasion wear, whereas that of sample B is adhesion wear. The improvement of wear performance for sample B is attributed to its ultrafine microstructure and attendant high hardness.
